# Laboratory Hints

**Published:** 1904-06

**Authors:** D. E. Seehan

**Affiliations:** Hamilton, Ohio.


### LABORATORY HINTS.

By D. E. Seehan, D. D. S., Hamilton, Ohio.

An Aid in Carrying Pumice.—When using felt cones or wheels in polishing rubber plates, gold crowns, etc., hold a piece of soap against the wet cone before applying the pumice. The soap on the cone prevents too much heat and will also carry the pumice.

To Keep a Supply of Soft Wax.—Punch a hole the size of your Bunsen burner in the bottom of a seamless salve-box. Push this down to a point just above the air-holes in the burner and solder in place. Fill box with scrap wax, and when the burner is in use you will always have an abundance of soft wax. With this convenience you will save the time usually consumed in picking up and balancing a piece of hard wax on the end of your spatula, in heating it.

To Prevent Bruising Plate When Swaging.—In swaging metal plates, that you will not bruise your plate, get a rubber tip usually used on crutches. Put this on the large end of your horn mallet and proceed.

A Soldering Hint.—When you solder your bridge, first of all have the investment hot. When using the blowpipe do not blow a strong blast, but blow gently enough to have a yellow streak in the flame from the pipe to the piece to be soldered. The yellow streak is essential. You can drag the soldier with a piece of strengthening bar stuck in the end of a pine stick.

To Burnish Backing Over the Edges of Teeth.—In backing up facings for bridges and crowns with heavy plate, especially cuspids, it is sometimes hard to burnish the backing over the incisive edge. To facilitate matters split the backing in several places parallel with the long axis of the tooth to the line where the plate is to bend. It will then burnish over easily. Go over this surface once with a file and it will hug the tooth perfectly.

Rapid Repair.—For rapidly repairing a plate when one or two teeth are broken out, dovetail the plate back of the pins set in the tooth and pack in a quick-setting amalgam. It will hold as well as a rubber patch.

Care of Seamless Crown Outfit.—Before putting away your seamless crown outfit after using, paint the holes in the draw-plate with oil. This prevents rusting of the plate, and the oil prevents stretching and consequent thinning of the gold in your crowns. Never drive gold through dry holes.—*Summary.*

